# 预测肺浸润性非黏液腺癌IASLC分级：基于双能CT成像及传统特征的列线图

**DOI:** 10.3779/j.issn.1009-3419.2025.106.24

**Published:** 2025-08-20

**Authors:** Kaibo ZHU, Liangna DENG, Yue HOU, Lulu XIONG, Caixia ZHU, Haisheng WANG, Junlin ZHOU

**Affiliations:** ^1^730030 兰州，兰州大学第二医院放射科，兰州大学第二临床医学院，甘肃省医学影像重点实验室，医学影像人工智能甘肃省国际科技合作基地（朱凯博，熊璐璐，王海升，周俊林）; ^1^Department of Radiology, Lanzhou University Second Hospital, the Second Clinical Medical School, Lanzhou University, Key Laboratory of Medical Imaging of Gansu Province, Gansu International Scientific and Technological Cooperation Base of Medical Imaging Artificial Intelligence, Lanzhou 730030, China; ^2^210009 南京，南京医科大学附属肿瘤医院放射科（邓靓娜）; ^2^Department of Radiology, The Affiliated Cancer Hospital of Nanjing Medical University, Nanjing 210009, China; ^3^730030 兰州，兰州大学第二医院呼吸内科（侯悦）; ^3^Department of Respiratory Medicine, Lanzhou University Second Hospital, Lanzhou 730030, China; ^4^730300 兰州，甘肃省医疗器械检验检测所，甘肃省医疗器械行业技术中心（朱彩霞）; ^4^Gansu Medical Device Inspection and Testing Institute, Gansu Province Medical Device Industry Technology Center, Lanzhou 730300, China

**Keywords:** 肺肿瘤, 浸润性非黏液腺癌, 能谱计算机断层扫描, 国际肺癌研究协会分级, 列线图, Lung neoplasms, Invasive non-mucinous pulmonary adenocarcinomas, Spectral computed tomography, International Association for the Study of Lung Cancer classification, Nomogram

## Abstract

**背景与目的:**

肺腺癌是非小细胞肺癌（non-small cell lung cancer, NSCLC）重要的病理组织学亚型。而肺浸润性非黏液腺癌（invasive non-mucinous pulmonary adenocarcinomas, INMA）因其显著异质性及组织学成分多样性，患者预后往往较差。建立INMA组织学分级系统是评价其恶性程度的关键。2021年国际肺癌研究协会（International Association for the Study of Lung Cancer, IASLC）提出新的组织学分级系统可以更好地对INMA患者进行预后分层。本研究旨在通过双能计算机断层扫描（dual-energy computed tomography, DECT）参数、分形维数（fractal dimension, FD）、临床特征及常规CT参数建立可视化列线图模型来术前预测INMA IASLC分级。

**方法:**

回顾性纳入2021年3月至2025年1月术前行DECT的INMA患者112例。根据IASLC分级将患者分为低-中级别组和高级别组。收集患者临床特征及常规CT参数，包括基线特征、生化标志物及血清肿瘤标志物。双能CT衍生参数，包括碘浓度（iodine concentration, IC）、有效原子序数（effective atomic number, eff-Z）和标准化碘浓度（normalized iodine concentration, NIC），采集并测定NIC比（NIC ratio, NICr）和FD。采用单因素分析比较两组在传统特征及双能CT衍生参数上的差异，将有统计学意义的变量纳入多因素*Logistic*回归分析，建立临床资料、常规CT参数及双能CT衍生参数的列线图模型并筛选INMA IASLC分级的独立预测因子；利用受试者工作特征（receiver operating characteristic, ROC）曲线分析评估判别能力。

**结果:**

多因素分析显示吸烟史[优势比（odds ratio, OR）=2.848, *P*=0.041]、分叶征（OR=2.163, *P*=0.004）、支气管充气征（OR=7.833, *P*=0.005）、动脉期eff-Z（OR=4.266, *P*<0.001）及动脉期IC（OR=1.290, *P*=0.012）是预测INMA IASLC分级的独立影响因素，基于上述指标构建的列线图模型预测性能最佳，曲线下面积（area under the curve, AUC）达0.804（95%CI: 0.725-0.883），特异度和灵敏度分别为85.3%和65.7%。

**结论:**

基于临床特征、影像学特征及能谱CT衍生参数的列线图模型在INMA IASLC分级的术前无创评估中具有较大的应用潜力。

## Abstract (en)

**Background and objective:**

Lung adenocarcinoma is an important pathohistologic subtype of non-small cell lung cancer (NSCLC). Invasive non-mucinous pulmonary adenocarcinomas (INMA) tend to have a poor prognosis due to their significant heterogeneity and diverse histologic components. Establishing a histologic grading system for INMA is crucial for evaluating its malignancy. In 2021, the International Association for the Study of Lung Cancer (IASLC) proposed that a new histological grading system could better stratify the prognosis of INMA patients. The aim of this study was to establish a visualized nomogram model to predict INMA IASLC grading preoperatively by means of dual-energy computed tomography (DECT), fractal dimension (FD), clinical features and conventional CT parameters.

**Methods:**

A total of 112 patients with INMA who underwent preoperative DECT were retrospectively enrolled from March 2021 to January 2025. Patients were categorized into low-intermediate grade and high grade groups based on IASLC grading. The clinical characteristics and conventional CT parameters, including baseline features, biochemical markers, and serum tumor markers, were collected. DECT-derived parameters, including iodine concentration (IC), effective atomic number (eff-Z), and normalized IC (NIC), were collected and determined as NIC ratio (NICr) and fractal dimension (FD). Univariate analysis was employed to compare differences in conventional characteristics and DECT parameters between the two groups. Variables demonstrating statistical significance were subsequently incorporated into a multivariate *Logistic *regression analysis. A nomogram model integrating clinical data, conventional CT parameters, and DECT parameters was developed to identify independent predictors for IASLC grading of INMA. The discriminatory performance of the model was evaluated using receiver operating characteristic (ROC) curve analysis.

**Results:**

Multivariate analysis identified smoking history [odds ratio (OR)=2.848, *P*=0.041], lobulation sign (OR=2.163, *P*=0.004), air bronchogram (OR=7.833, *P*=0.005), eff-Z in arterial phase (OR=4.266, *P*<0.001), and IC in arterial phase (OR=1.290, *P*=0.012) as independent and significant predictors for IASLC grading of INMA. The nomogram model constructed based on these indicators demonstrated optimal predictive performance, achieving an area under the curve (AUC) of 0.804 (95%CI: 0.725-0.883), with specificity and sensitivity of 85.3% and 65.7%, respectively.

**Conclusion:**

The nomogram model based on clinical features, imaging features and spectral CT parameters have a large potential for application in the preoperative noninvasive assessment of INMA IASLC grading.

肺浸润性非黏液腺癌（invasive non-mucinous adenocarcinoma, INMA）是较为常见的肺癌组织学类型，因其显著异质性及组织学成分多样性，患者预后往往较差^[1]^。2015年世界卫生组织（World Health Organization, WHO）第4版肺肿瘤分类依据主要生长模式将INMA分为3级^[2]^，然而该分类系统可能忽略了INMA的组织学异质性，低估了非优势组织学亚型的影响^[3]^。2021年，国际肺癌研究协会（International Association for the Study of Lung Cancer, IASLC）提出了一种新的分级方法，该体系综合主要生长模式与高级别成分占比以确定INMA的分化等级。研究^[4,5]^证实IASLC分级系统能为术前辅助治疗和手术方案选择的获益评估提供关键指导，同时更精准地预测患者预后。

尽管术前活检能够获得IASLC分级，但因其侵入性、术前活检标本不能完全反映病变的生物学特性及气胸和出血等并发症，其诊断准确性受到限制^[6]^。因此，迫切需要寻找一种无创、方便有效的INMA分级术前鉴别方法。双能计算机断层扫描（dual-energy computed tomography, DECT）是一种新型功能成像设备^[7]^，与传统CT设备相比，DECT可以获得客观、准确地反映病灶内部组织学和生物学特征的额外信息及血流动力学相关信息的多种定量参数^[8,9]^，如有效原子序数（effective atomic number, eff-Z）、碘浓度（iodine concentration, IC）及标准化IC（normalized IC, NIC）等。碘图基于特定的线性衰减系数计算，不受金属伪影等干扰因素的影响，可确保提供准确的定量参数。既往CT能谱定量分析的相关研究^[10,11]^已在肿瘤诊断、鉴别及疗效评估等方面取得了重要进展。目前尚无研究通过DECT从原发病中获取定量参数来预测INMA IASLC分级。

分型分析是一种量化自然存在的且具有高度复杂结构物体的研究方法^[12]^。分形维数（fractal dimension, FD）是其用于描述物体复杂性、不规则性和分布特点的重要参数。FD分析近年来广泛应用于中枢神经系统影像分析，肺实体肿瘤领域研究相对较少^[13]^。考虑到DECT和FD分析在肺肿瘤中的临床价值、实用性及可重复性等优势。本研究旨在探讨FD、DECT定量参数、临床因素及常规CT特征是否可以术前预测INMA患者IASLC分级，并构建可视化的诊断模型，以辅助临床手术前的治疗决策。

## 1 资料与方法

### 1.1 研究人群

本研究经兰州大学第二医院伦理委员会批准（No.2025A-563），并豁免知情同意。回顾性筛选2021年3月至2025年1月兰州大学第二医院经手术病理证实的INMA患者196例，严格遵循纳入和排除标准，研究最终纳入符合标准的INMA患者112例，其中男性66例，女性46例。纳入标准：（1）经手术病理证实为INMA；（2）患者临床资料和影像资料完整；（3）患者术前行胸部DECT检查，且扫描时间与手术时间间隔小于2周。排除标准：（1）病灶表现为混合磨玻璃结节或亚实性结节；（2）最大径<10 mm的病变；（3）术前接受抗肿瘤治疗；（4）微浸润性腺癌、浸润性黏液腺癌及其他腺癌亚型（胶样型、肠型及胎儿型等）；（5）能谱图像伪影太重。通过医院影像归档和通讯系统（picture archiving and communication system, PACS）收集患者的性别、吸烟史、实验室检查及生化检查资料。本研究的患者入组流程图见图1。

**图1:**
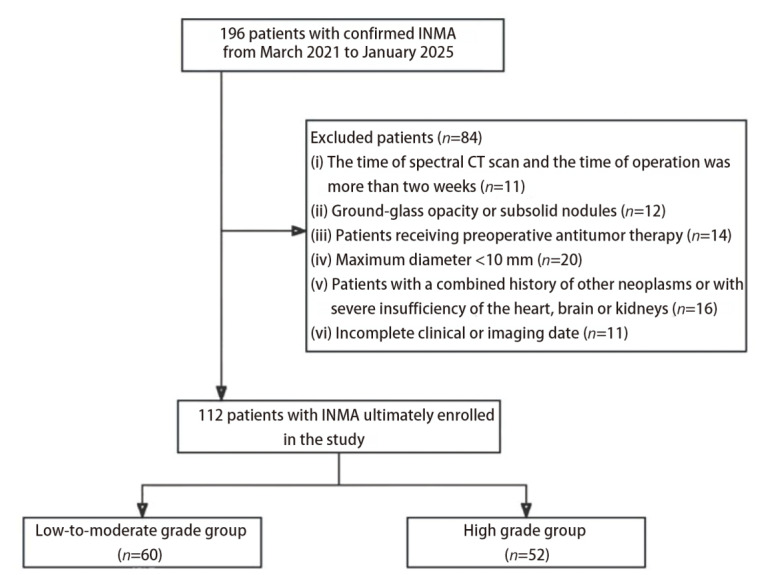
研究的患者入组流程图

### 1.2 组织病理学评估

所有患者均行肺肿瘤切除术，病理标本取样，石蜡包埋切片，苏木精-伊红（hematoxylin-eosin, HE）染色。由2位具有6年以上肺部诊断经验的病理学家在显微镜下对112例参与者的HE染色玻片进行重新评估，对有歧义的病理组织标本通过讨论达成共识。新的IASLC分级系统基于主要亚型和高级别成分超过20%将腺癌分为3个级别，其中高级别成分包括实体型、微乳头型、筛孔型和复杂腺体型等，相比I和II级患者，III级INMA患者预后更差，因此本研究将患者分为低-中级别（即I和II级）组和高级别（III级）组^[14]^，具体分级见IASLC对可切除INMA分级系统。

### 1.3 CT检查方案

所有患者均接受术前DECT检查，使用通用电气（general electric, GE）医疗Discovery CT 750 HD和Revolution CT设备进行全胸宝石能谱成像模式（gemstone spectral imaging, GSI）扫描。患者取标准仰卧位，双臂上举以降低呼吸伪影干扰，扫描范围完整覆盖肺野，上起胸廓入口，下达肋膈角。双能瞬时切换（140/80 kVp，切换时间0.5 min），智能毫安调制（Smart mA），探测器准直宽度为0.625 mm，机架旋转周期0.7 s/rot，螺距因子0.984:1。扫描采用5 mm层厚连续采集，原始数据经迭代重建（ASiR-V 50%）生成1.25 mm薄层图像，层间距1.25 mm。经肘前静脉以3-4 mL/s流速（剂量1.2 mL/kg）团注碘海醇（Ultravist 300, Bayer Schering Pharma, Berlin, Germany），采用主动脉阈值触发技术（监测层面：隆突水平胸主动脉，阈值80 HU），于触发后8 s[动脉期（arterial phase, AP）]及30 s[静脉期（venous phase, VP）]行双期GSI扫描。所有重建图像自动传输至Advantage Workstation 4.7工作站（AW 4.7）进行后处理分析。

### 1.4 图形分析和数据测量

由1名具有5年胸部影像诊断经验的放射科医师独立完成临床数据采集及CT影像学评估。基于PACS系统（Carestream Vue PACS 12.1, Carestream Health）及GSI Viewer后处理平台（AW 4.7, GE Healthcare）评估肿瘤形态学特征，其中包括在肺窗及纵隔窗上判定肿瘤的影像学特征，存在疑问时求助高年资主任医师解决。DECT分析AP及VP图像时，在肿瘤最大横截面积层面上选择实性成分区域，尽量避开血管、坏死及钙化区域，勾画感兴趣区域（region of interest, ROI），确保双期ROI位置保持一致，并在相邻连续3层进行参数测量取均值。同步测量胸主动脉ROI，计算NIC（NIC=病灶IC/主动脉IC）、能谱曲线斜率K值[K=(CT40 keV-CT90 keV)/(90-40)]及动静脉期NIC比值（NICr=NICa/NICv）。

为评估测量可重复性，随机抽取50%病例（*n*=56）由第二位放射科医师（7年工作经验）重复上述流程，采用Bland-Altman分析法（一致性界限=均值±1.96SD）及组内相关系数（intraclass correlation coefficient, ICC）来评价观察者间信度（ICC>0.75判定为良好一致性）。

### 1.5 分形维数分析

将患者的术前胸部CT原始DICOM数据导入ITK-SNAP软件（版本3.8.0），由具有6年胸部影像诊断经验的放射科医师在轴位肺窗（窗宽1500 HU，窗位-500 HU）下选取肿瘤最大横截面积层面。随后通过手动分割工具勾画INMA病灶边界生成初始ROI，所有ROI均经另一位资深胸部影像专家（10年经验）进行盲法审核并确认空间一致性后保存。将质控后的ROI图像导入ImageJ平台（版本1.54d；美国国立卫生研究院）进行预处理：先将图像标准化为8位灰度格式，继而通过Fraclac插件（版本2.5）提取形态学特征并计算FD，最终通过3次独立重复测量取均值作为FD定量结果。在本研究中，使用盒计数法，公式如下：FD=KL-NL，其中L代表盒的大小，NL代表覆盖研究对象所需尺寸，Log K代表线性回归（NL与L）获得的Y轴截距。。

### 1.6 统计学分析

采用统计软件SPSS 26.0（IBM Corp, Armonk, NY, USA）、R软件（4.2.6版）及MedCalc（20.2.6版）进行统计分析。通过ICC选择双人盲法评估来验证两位放射科医师所获DECT参数的一致性（ICC>0.75判定为可接受信度）。连续性变量经*Shapiro-Wilk*检验评估后，符合正态分布的数据使用均数±标准差描述，组间差异比较选用独立样本*t*检验；不符合正态分布的数据使用中位数和四分位数间距描述，使用*Mann-Whitney U*检验比较组间差异。分类变量使用例数（百分比）描述，组间比较采用χ²检验或*Fisher*精确概率法。通过逐步向后法多因素 *Logistic*回归筛选独立预测因子并构建预测模型，以列线图展示。模型性能通过受试者工作特征（receiver operating characteristic, ROC）曲线下面积（area under the curve, AUC）、校准曲线及决策曲线分析（decision curve analysis, DCA）评估，由后者量化净获益阈值范围。*P*<0.05为差异具有统计学意义。

## 2 结果

### 2.1 临床特点

低-中级别组和高级别组患者的基线特征见表1，两组患者在吸烟史方面差异有统计学意义（*P*=0.005）；在年龄、性别、增殖指数（antigen Ki-67, Ki-67）、肿瘤标志物[癌胚抗原（carcinoembryonic antigen, CEA）、神经元特异性烯醇化酶（neuron-specific enolase, NSE）、细胞角蛋白片段19（cytokeratin 19 fragment antigen 21-1, CYFRA21-1）、胃泌素前释放肽（progastrin releasing peptide, ProGRP）、鳞状细胞癌抗原（squamous cell careinoma antigen, SCCA）]及生化标记物[血小板计数（platelet count, PLT）、中性粒细胞计数（neutrophil count, NEUT）、淋巴细胞计数（lymphocyte count, LYMPH）及中性粒细胞计数/淋巴细胞计数（NEUT/LYMPH）]等方面，差异均无统计学意义（*P*>0.05）。

**表1:** 112例INMA患者的基线临床资料

Variables	Total (*n*=112)	Low-to-moderate grade group (*n*=60)	High grade group (*n*=52)	*t/χ*²*/Z*	*P*
Gender, *n* (%)				0.019	0.891
Female	46 (41.071)	25 (41.667)	21 (40.385)		
Male	66 (58.929)	35 (58.333)	31 (59.615)		
Age (yr)	57.500 (53.000, 65.000)	56.500 (52.000, 65.000)	58.500 (55.000, 66.000)	-0.867	0.386
Smoking history, *n* (%)				2.617	0.005
No	80 (71.429)	39 (65.000)	41 (78.846)		
Yes	32 (28.571)	21 (35.000)	11 (21.154)		
Ki-67 (%)	30.000 (20.000, 51.250)	30.000 (13.750, 40.000)	30.000 (13.750, 40.000)	-2.611	0.090
CEA, *n* (%)				0.597	0.440
Normal (-)	71 (63.393)	40 (66.667)	31 (59.615)		
High (+)	41 (36.607)	20 (33.333)	21 (40.385)		
NSE, *n* (%)				0.140	0.708
Normal (-)	90 (80.357)	49 (81.667)	41 (78.846)		
High (+)	22 (19.643)	11 (18.333)	11 (21.154)		
CYFRA21-1, *n* (%)				0.735	0.391
Normal (-)	88 (78.571)	49 (81.667)	39 (75.000)		
High (+)	24 (21.429)	11 (18.333)	13 (25.000)		
ProGRP, *n* (%)				0.342	0.559
Normal (-)	95 (84.821)	52 (86.667)	43 (82.692)		
High (+)	17 (15.179)	8 (13.333)	9 (17.308)		
SCCA, n (%)				0.462	0.790
Normal (-)	108 (96.429)	58 (96.667)	50 (96.154)		
High (+)	4 (3.571)	2 (3.333)	2 (3.846)		
PLT (×10^9^/L)	196.000 (156.000, 235.250)	196.000 (150.000, 235.250)	199.500 (161.500, 234.500)	-0.242	0.809
NEUT (×10^9^/L)	3.520 (2.678, 4.982)	3.305 (2.678, 4.630)	3.675 (2.735, 5.650)	-1.009	0.313
LYMPH (×10^9^/L)	1.460 (1.052, 1.950)	1.430 (1.027, 1.917)	1.480 (1.095, 1.987)	-0.105	0.916
NEUT/LYMPH	2.170 (1.492, 4.095)	2.175 (1.367, 3.608)	2.170 (1.652, 4.535)	-0.665	0.506

*t*: *t*-test; *Z*: *Mann-Whitney U* test; *χ*²: *Chi-square* test; CEA: carcinoembryonic antigen; NSE: neuron-specific enolase; CYFRA21-1: cytokeratin fragment 19; ProGRP: progastrin-releasing peptide; SCCA: squamous cell carcinoma antigen; PLT: platelet count; NEUT: neutrophil count; LYMPH: lymphocyte count.

### 2.2 常规CT特征

低-中级别组和高级别组患者常规CT上原发病变形态学特征的比较见表2，从位置、大小、平均CT密度值、边界清晰、分叶征、毛刺征、胸膜凹陷征、血管集束征、支气管充气征及空泡征分析两组INMA患者的CT表现特征。INMA好发于右肺上叶（33/112）；两组在分叶征、胸膜凹陷征和支气管充气征方面差异有统计学意义（*P*=0.035、*P*=0.036和*P*=0.013），而位置、大小、平均CT密度值、边界清晰、毛刺征、血管集束征及空泡征等差异均无统计学意义（*P*>0.05）。

**表2:** 低-中级别组和高级别组INMA的影像特征比较

Variables	Total (*n*=112)	Low-to-moderate grade group (*n*=60)	High grade group (*n*=52)	t/*χ*²	*P*
Size (mm)	25.120±8.678	24.412±8.294	25.939±9.112	-0.928	0.355
Density (HU)	35.564±13.435	34.967±15.055	36.254±11.389	-0.514	0.608
Lobe location, *n* (%)				2.462	0.651
Right upper	33 (29.464)	20 (33.333)	13 (25.000)		
Right middle	7 (6.250)	5 (8.333)	2 (3.846)		
Right lower	24 (21.429)	11 (18.333)	13 (25.000)		
Left upper	23 (20.536)	12 (20.000)	11 (21.154)		
Left lower	25 (22.321)	12 (20.000)	13 (25.000)		
Clear boundary, *n* (%)				3.291	0.070
Yes	63 (56.250)	29 (48.333)	34 (65.385)		
No	49 (43.750)	31 (51.667)	18 (34.615)		
Lobulation sign, *n* (%)				7.364	0.035
No	52 (46.429)	35 (58.333)	17 (32.692)		
Yes	60 (53.571)	25 (41.667)	35 (67.308)		
Spiculation, *n* (%)				0.620	0.431
No	23 (20.536)	14 (23.333)	9 (17.308)		
Yes	89 (79.464)	46 (76.667)	43 (82.692)		
Pleural retraction, *n* (%)				6.509	0.036
No	25 (22.321)	19 (31.667)	6 (11.538)		
Yes	87 (77.679)	41 (68.333)	46 (88.462)		
Vascular convergence, *n* (%)				0.319	0.572
No	72 (64.286)	40 (66.667)	32 (61.538)		
Yes	40 (35.714)	20 (33.333)	20 (38.462)		
Air bronchogram, *n* (%)				6.837	0.013
No	84 (75.000)	48 (80.000)	36 (69.231)		
Yes	28 (25.000)	12 (20.000)	16 (30.769)		
Bubblelike lucency, *n* (%)				1.318	0.251
No	78 (69.643)	39 (65.000)	39 (75.000)		
Yes	34 (30.357)	21 (35.000)	13 (25.000)		

### 2.3 DECT参数和FD分析

低-中级别组与高级别组DECT参数差异见表3。INMA高级别组中动脉期的eff-Z（*P*=0.001）、IC（*P*<0.001）、NIC（*P*=0.001）、K值（*P*<0.001）和静脉期IC（*P*=0.005）、NIC（*P*=0.026）均低于低-中级别组；INMA高级别组FD高于低-中级别组，差异有统计学意义（*P*=0.012）。而NICr（*P*=0.085）、静脉期eff-Z（*P*=0.126）和静脉期K值（*P*=0.731）差异无统计学意义（*P*>0.05）。图2和图3分别为INMA高级别组和低-中级别组的代表示例。Bland-Altman图提示动脉期和静脉期的IC、NIC、eff-Z及K值在评估的观察者间显示出良好的一致性。DECT参数的ICC值为0.801-0.974（表4）。

**图2:**
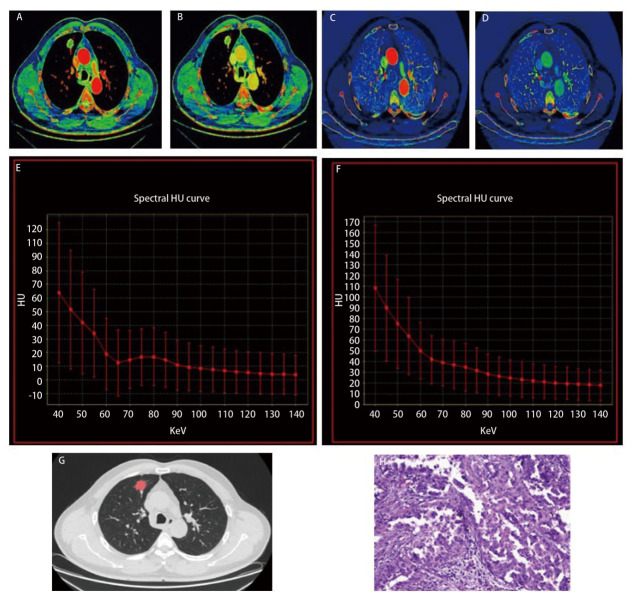
53岁男性患者，高级别INMA患者。 A、B：AP和VP的有效原子序数（eff-Z分别为8.09和8.16）；C、D：AP和VP IC伪彩图像（IC分别为7.85和8.93 mg/cm^3^）；E、F：AP和VP的光谱曲线图像（K分别为1.06和1.21）；G：肺窗的分形维数分析，分形维数为1.67；H: 苏木精-伊红染色（×100）。

**图3:**
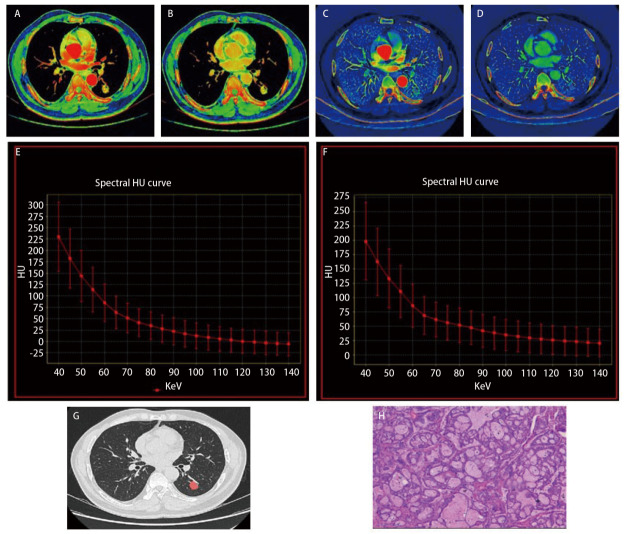
50岁男性患者，低-中级别组INMA患者。 A、B：AP和VP的有效原子序数（eff-Z分别为9.38和9.03）；C、D：AP和VP IC伪彩图像（IC分别为31.63和24.82 mg/cm^3^）；E、F：AP和VP的光谱曲线图像（K分别为3.62和2.98）；G：肺窗的分形维数分析，分形维数为1.74；H：苏木精-伊红染色（×100）。

**表3:** 低-中级组和高级别组INMA的光谱参数比较

Variables	Total (*n*=112)	Low-to-moderate grade group (*n*=60)	High grade group (*n*=52)	*t*/Z	*P*
IC in AP (mg/cm^3^)	17.455±8.580	20.904±8.252	14.466±7.750	-4.255	<0.001
eff-Z in AP	8.555 (8.287, 8.922)	8.715 (8.450, 9.098)	8.460 (8.133, 8.803)	-3.194	0.001
NIC in AP (mg/mL)	0.135±0.061	0.155±0.060	0.118±0.058	-3.283	0.001
K in AP	2.368±1.167	2.838±1.121	1.961±1.054	-4.263	<0.001
IIC in VP (mg/cm^3^)	15.916±7.925	18.134±8.783	13.993±6.588	-2.844	0.005
eff-Z in VP	8.484±0.372	8.434±0.363	8.542±0.377	-1.541	0.126
NIC in VP (mg/mL)	0.375 (0.297, 0.490)	0.400 (0.328, 0.550)	0.350 (0.247, 0.455)	-2.226	0.026
K in VP	0.495 (0.307, 1.030)	0.570 (0.268, 0.900)	0.445 (0.367, 1.172)	-0.344	0.731
NICr	0.375±0.183	0.347±0.140	0.408±0.219	-1.740	0.085
FD	1.672±0.080	1.655±0.082	1.692±0.074	-2.554	0.012

IC: iodine concentration; eff-Z: effective atomic number; NIC: normalized IC; NICr: NIC ratio; FD: fractal dimension; AP: arterial phase; VP: venous phase; K: slope of energy spectrum curve.

**表4:** ICC的DECT相关参数

Parameters	ICC (95%CI)
IC in AP	0.801 (0.683-0.878)
eff-Z in AP	0.883 (0.809-0.930)
NIC in AP	0.891 (0.821-0.935)
K in AP	0.953 (0.922-0.972)
IC in VP	0.932 (0.887-0.960)
eff-Z in VP	0.894 (0.826-0.937)
NIC in VP	0.974 (0.956-0.985)
K in VP	0.962 (0.937-0.978)

ICC: intragroup correlation coefficient; DECT: dual-energy computed tomography; CI: confidence interval.

### 2.4 个体化预测模型的建立

将单因素分析有统计学意义的变量纳入多因素*Logistic*回归分析探讨临床-影像学预测模型的独立预测因子（表5），结果显示吸烟史[优势比（odds ratio, OR）=2.848, 95%CI: 1.024-8.000, *P*=0.041]、分叶征（OR=2.163, 95%CI: 1.476-5.335, *P*=0.004）、支气管充气征（OR=7.833, 95%CI: 1.837-33.396, *P*=0.005）、动脉期eff-Z（OR=4.266, 95%CI: 1.746-10.422, *P*<0.001）及动脉期IC（OR=1.290, 95%CI: 1.049-1.781, *P*=0.012）与INMA IASLC分级有关。

**表5:** INMA IASLC分级的独立危险因素

Variables	*β*	SE	OR (95%CI)	Z	P
Air bronchogram	2.058	0.740	7.833 (1.837-33.396)	2.782	0.005
IC in AP	0.255	0.165	1.290 (1.049-1.781)	1.549	0.012
Lobulation sign	0.771	0.461	2.163 (1.476-5.335)	1.672	0.004
eff-Z in AP	1.451	0.456	4.266 (1.746-10.422)	3.183	<0.001
Smoking history	1.047	0.527	2.848 (1.024-8.000)	1.987	0.041

IASLC: International Association for the Study of Lung Cancer; OR: odds ratio; SE: standard error.

基于上述独立预测因子构建列线图模型见图4A，该列线图具有最佳的识别能力，AUC为0.804（95%CI: 0.725-0.883）（图4B，表6）；Hosmer-Lemeshow校准曲线（图4C）显示，该模型预测INMA的IASLS分级与实际情况吻合较好；决策曲线（图4D）显示了可以从该列线图模型中获得净收益的阈值概率。

**图4:**
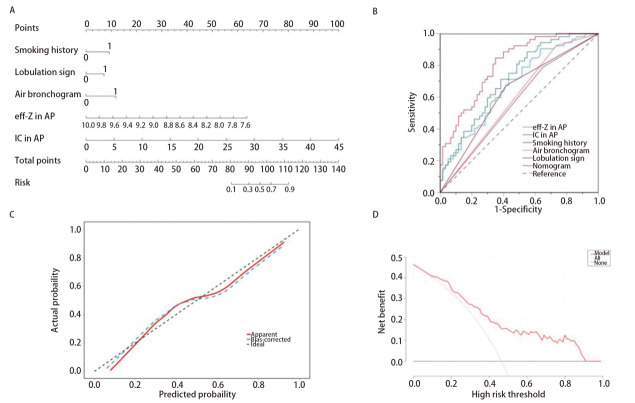
预测INMA IASLC分级的列线图模型的构建、效能评估与验证。 A: 预测INMA IASLC分级的列线图，用吸烟史、分叶征、支气管充气征、动脉期eff-Z及IC绘制；B: 各预测因子的ROC曲线评估和比较列线图模型的诊断效能；C、D：列线图模型预测INMA IASLC分级的校准曲线和决策曲线。

**表6:** 独立危险因素与组合模型的诊断效能

Parameters	AUC (95%CI)	Sensitivity (95%CI)	Specificity (95%CI)	Cut off
Smoking history	0.569 (0.463-0.675)	0.357 (0.233-0.475)	0.791 (0.680-0.902)	0.428
Lobulation sign	0.628 (0.524-0.732)	0.586 (0.464-0.712)	0.677 (0.556-0.803)	0.455
Air bronchogram	0.595 (0.490-0.700)	0.275 (0.156-0.384)	0.920 (0.847-0.963)	0.361
eff-Z in AP	0.676 (0.577-0.774)	0.571 (0.441-0.686)	0.714 (0.594-0.831)	8.505
IC in AP	0.715 (0.621-0.808)	0.452 (0.320-0.582)	0.881 (0.801-0.968)	12.075
Combination model	0.804 (0.725-0.883)	0.657 (0.533-0.772)	0.853 (0.751- 0.937)	0.348

AUC: area under the curve.

## 3 讨论

含高级别亚型的INMA患者预后往往较差，术前准确识别IASLC分级对临床治疗决策的选择和预后具有重要价值。本研究创新性地整合FD分析、临床因素、常规CT征象及DECT参数，构建了INMA的IASLC分级预测模型。与基于图像内部灰度和纹理的影像组学不同，FD能够描述肿瘤组织的力学参数及整体生长形态，其可重复性和稳定性更优^[12]^。DECT在不改变常规工作流程、不增加辐射剂量的前提下，可以提供多参数定量分析^[7]^，为临床治疗选择决策提供了更为科学的依据。

既往研究^[15,16]^证实，有吸烟史的肺腺癌患者往往表现出更高的肿瘤实性成分比例及更强的侵袭性，这种差异可能与吸烟诱导的慢性炎症微环境、DNA修复机制受损及持续性氧化应激损伤密切相关。本研究也证实了吸烟史是IASLC分级的独立危险因素，有吸烟史的INMA患者比无吸烟史的患者IASLC分级更高。

在常规CT征象中典型恶性征象，如毛刺征、分叶征及胸膜凹陷征等出现的频率与结节恶性程度呈正相关^[17-20]^。值得注意的是，本研究发现分叶征、胸膜凹陷征及支气管充气征的出现频率与病理级别呈正向关联（*P*<0.05），而空泡征和血管集束征则无此趋势。分叶征形似多个结节融合，多由于肿瘤细胞在各个方向的无序分裂和增殖存在差异，也可能与周围肺组织对其生长的阻碍有关^[21]^，提示INMA IASLC高级别组表达时肿瘤浸润程度较高，反映肿瘤快速生长及侵袭性。也有研究^[22,23]^证实分叶征对肺腺癌演进谱系（AIS-MIA-IAC）具有良好的鉴别价值。此外，支气管充气征常与分叶征、毛刺征、胸膜凹陷征及空泡征等其他影像特征同时存在，该征象在鉴别浸润前病变和浸润性病变中具有重要意义，Liu等^[24]^认为出现支气管充气征更支持浸润性病变的诊断。本研究将分叶征、胸膜凹陷征和支气管充气征纳入多因素*Logistic*回归分析，发现分叶征和支气管充气征可以作为划分危险程度的指标，当肿瘤出现分叶征和支气管充气征时，病理诊断更倾向于高级别组。

IC、NIC和eff-Z由DECT衍生而来，能够反映肿瘤的血流动力学信息并提示肿瘤细胞的增殖情况^[25]^，本研究表明低-中级别组INMA患者的动脉期IC和eff-Z水平显著高于高级别组，是INMA IASLC分级的独立预测因子。IC可间接反映肿瘤在特定阶段的组织灌注水平及血管化程度，即IC值越高则肿瘤血供越丰富^[26]^，Deng等^[27]^认为双能CT分析中-低级别肿瘤的IC与NIC显著高于高级别肿瘤，本研究同样证实含高级别亚型的INMA血供更差。这一现象可能归因于高级别肿瘤虽表现出血管生成活跃的特性，但其新生血管结构紊乱且生长速度过快，导致中心坏死概率显著增加，同时伴随血管通透性异常升高，削弱了碘剂在病灶内的有效蓄积。eff-Z是具有与化合物或混合物相同衰变系数的元素的原子序数，常用于反映物质的组织成分。已有研究^[28,29]^证实eff-Z在肿瘤鉴别诊断、淋巴结转移预测以及病灶稳定性评估中具有重要临床价值，本研究发现动脉期eff-Z与高级别INMA呈负向相关，其潜在的组织病理学基础在于高级别INMA中更常见坏死及囊变区域，拉低了整个病灶的动脉期eff-Z平均测量值；此外，可能受碘对比剂分布影响，动脉期eff-Z部分较低反映了高级别肿瘤内部坏死区无强化的特点或对比剂渗透障碍^[26]^。

FD作为一种量化肿瘤形态复杂性和异质性的参数，现已被证明是评估肿瘤复杂性的有效工具，近年来在实体肿瘤研究中展现出重要价值^[[30,31]^。Sánchez等^[32]^通过分析脑肿瘤的形态学和分形特征，指出不同肿瘤间表面复杂性和分形特征存在差异；Deng等^[27]^利用DECT及FD分析预测N0期NSCLC患者淋巴血管浸润（lymphovascular invasion, LVI），结果表明FD是预测N0期NSCLC LVI的独立影响因素，能够反映肿瘤的恶性程度及预后。本研究结果显示，高级别INMA患者的FD高于低-中级别INMA患者（*P*<0.05），可能与侵袭性增加或基因突变，如Kirsten大鼠肉瘤病毒癌基因同源物（Kirsten rats arcomaviral oncogene homolog, *KRAS*）基因突变有关，进而增加了肿瘤组织的形态复杂性和不规则性，如出现分叶征、毛刺征及支气管充气征等影像学征象，以上变化均能通过观察FD值的变化来客观反映。此外，由于*KRAS*等突变往往与患者生存期密切相关，因此FD分析也间接反映患者的预后，这与既往研究^[27,33]^基本一致。本研究中FD在INMA IASLC低-中级组和高级组之间差异有统计学意义（*P*<0.05），遗憾的是，受样本量较少、肿瘤的其他特征及瘤内异质性等诸多因素影响，本研究中FD分析在预测INMA IASLC分级中的作用有限。

本研究存在一定的局限性。首先，尽管采用严格的纳入和排除标准，但回顾性设计固有的选择偏倚仍可能影响结论；其次，单中心样本量有限（*n*=112），尤其亚组分析时统计效能可能不足，未来需通过多中心、前瞻性队列研究扩大样本量并校正混杂因素，比如表皮生长因子受体（epidermal growth factor receptor, *EGFR*）突变状态。最后，FD计算基于肿瘤最大横截面单层面ROI，虽具有临床可操作性，但可能会低估肿瘤异质性，未来可探索基于人工智能自动分割的三维容积分析以优化FD量化策略。

综上所述，基于上述独立预测因子，本研究开发了个体化列线图模型，其校准曲线及决策曲线进一步证实了模型的临床适用性。当模型术前预测为低-中级别的患者，医生可更积极地选择肺段或楔形切除术，最大程度保留其肺功能和患者的生活质量。反之，预测为高级别的患者，则倾向于标准的肺叶切除术加系统性淋巴结清扫，以降低局部复发风险。IASLC分级是术后辅助治疗决策的关键因素之一，高级别常提示需要更积极的辅助治疗，本研究开发的模型为术前无创评估INMA侵袭潜能及个体化手术方案提供了可量化的决策支持工具。
